# Directional entry and release of Zika virus from polarized epithelial cells

**DOI:** 10.1186/s12985-019-1200-2

**Published:** 2019-08-08

**Authors:** Manasi Tamhankar, Jean L. Patterson

**Affiliations:** 1 Department of Microbiology, Immunology and Molecular Genetics, University of Texas Health San Antonio, San Antonio, TX USA; 2 Department of Virology and Immunology, Texas Biomedical Research Institute, San Antonio, TX USA

**Keywords:** Zika virus, Caco-2, Polarized cells, Egress, Permeability

## Abstract

**Background:**

Both vector borne and sexual transmission of Zika virus (ZIKV) involve infection of epithelial cells in the initial stages of infection. Epithelial cells are unique in their ability to form polarized monolayers and their barrier function. Cell polarity induces an asymmetry in the epithelial monolayer, which is maintained by tight junctions and specialized sorting machinery. This differential localization can have a potential impact of virus infection. Asymmetrical distribution of a viral receptor can restrict virus entry to a particular membrane while polarized sorting can lead to a directional release of virions. The present study examined the impact of cell polarity on ZIKV infection and release.

**Methods:**

A polarized Caco-2 cell model we described previously was used to assess ZIKV infection. Transepithelial resistance (TEER) was used to assess epithelial cell polarity, and virus infection was measured by immunofluorescence microscopy and qRT-PCR. Cell permeability was measured using a fluorescein leakage assay. Statistical significance was calculated using one-way ANOVA and significance was set at *p* < 0.05.

**Results:**

Using the Caco-2 cell model for polarized epithelial cells, we report that Zika virus preferentially infects polarized cells from the apical route and is released vectorially through the basolateral route. Our data also indicates that release occurs without disruption of cell permeability.

**Conclusions:**

Our results show that ZIKV has directional infection and egress in a polarized cell system. This mechanism of directional infection may be one of the mechanisms that enables the cross the epithelial barrier effectively without a disruption in cell monolayer integrity. Elucidation of entry and release characteristics of Zika virus in polarized epithelial cells can lead to better understanding of virus dissemination in the host, and can help in developing effective therapeutic interventions.

**Electronic supplementary material:**

The online version of this article (10.1186/s12985-019-1200-2) contains supplementary material, which is available to authorized users.

## Background

Originally discovered in Uganda in 1947, Zika virus (ZIKV) has recently emerged in the Americas to spread rapidly in Central and South American countries and has caused widespread outbreaks in Brazil [1–4]. While mosquito-borne transmission is the most common, other routes of transmission, including sexual transmission, have been reported [5, 6]. Studies of virus pathogenesis in endothelial cells and skin cells have been described [7, 8]. However, kinetics of peripheral dissemination has not been completely elucidated. Numerous studies show that the ZIKV is able to gain access to immune privilege sites like the testes and eyes [9, 10]. In addition, both clinical and animal model data show transplacental transmission of ZIKV has long term consequences for the fetus including microcephaly and other neurological defects [11, 12]. This ability of ZIKV to gain access to immune privilege sites points to the ability of the virus cross the permeability barrier to gain access to the tissue space and seems an important factor in the dissemination of the virus in the host.

Polarized cells differentially distribute lipids and proteins in the plasma membrane creating a distinct apical and basolateral surface [13, 14]. Tight junctions form a fence like barrier separating these apical and basolateral surfaces and render the cell monolayer selectively permeable to solutes and fluid [15, 16]. This requires specific targeting of ion channels, transporters and other accessory proteins to the two cell membranes [13]. This has important consequences during virus infection and dissemination. In order to establish infection, viruses have to invade the monolayer of epithelial cells [17–19]. Both the entry and the release of viruses may be polarized, and can take place selectively at either the apical or the basolateral membrane [20, 21]. Thus, receptors and other necessary entry factors may be differentially distributed at different membranes or even be inaccessible at one surface during infection. This lack of access can thus cause changes in cell susceptibility. Similarly, in addition to the normal sorting machinery, reports suggest that polarized epithelial cells have specific endosomal compartments that participate in specific apical of basolateral targeting [22, 23]. Viruses exploit this sorting pathway during infection which facilitate their delivery at a specific membrane for assembly and release [24]. This results into specific entry and egress kinetics in viruses with infection and budding being more efficient at one surface or another, and thus affecting virus dissemination in the host as a whole.

Caco-2 cells serve as an excellent model to study the permeability barrier since they readily form tight junctions when grown on a semipermeable barrier [25, 26]. Polarized Caco-2 cells have been used to investigate pathogenesis of a number of flaviviruses including, Japanese Encephalitis Virus (JEV) and Tick-borne encephalitis virus (TBEV)(−[27, 28]. In this study, we present evidence that infection of ZIKV occurs with greater efficiency on apical surface. Unlike other flaviviruses like TBEV or JEV, replication occurs without significant changes in paracellular permeability. Despite this, ZIKV is released vectorially through the basolateral route, indicating it’s an active transport across the epithelial barrier and not passive leakage. Thus, translocation may be one of the ways ZIKV crosses the tight junction barrier during dissemination in the host.

## Methods

### Cells and virus

Caco-2 cells (ATCC) were maintained in minimal essential medium (MEM; Invitrogen) supplemented with 2% or 10% fetal bovine serum (Invitrogen). Zika virus PR (Puerto Rico; GenBank KX087101.3; passage 3 and 4) was used for all the experiments and titers were determined with plaque assay performed on Vero-E6 cells.

### Transepithelial electrical resistance (TEER) measurements

4 × 10^4^ Caco-2 cells were seeded onto 6.5 mm diameter, 1 μm pore size polycarbonate membrane trans-wells (Costar) and media was replaced at 2 d intervals. Establishment of confluence was determined by measuring transepithelial electrical resistance (TEER) over the monolayer, using a Millicell-ERS volt-ohmmeter (Millipore, Billerica, MA).The electrodes of the epithelial volt-ohmmeter were first rinsed with 70% ethanol, followed by incubation in MEM without FBS supplementation for 10 mins at RT. All TEER measurements were made in a cell culture hood. Since temperature is known to affect resistance, measurements were made within 5 mins of removal of Transwells from the incubator [28]. Values were obtained by subtraction of a background value (i.e., TEER of filters without cell growth) and by multiplication by the area of the filter.

### Immunofluorescence microscopy

Caco-2 cells were seeded on polycarbonate transwell inserts (Corning; 6.5 mm; 3.0 μM pore size) and infected with 3 plaque forming unit (pfu)/ cell ZIKV. After infection, cells were fixed with 10% PBS buffered formalin and processed for immunofluorescence as described with some modifications (http://www.zonapse.net/protocols/id6.html). Briefly, Culture inserts were fixed in 10% buffered formalin overnight followed by washing with PBS. Cells were equilibriated with IMF buffer (20 mM HEPES, pH 7.5, 0.1% Triton-X-100, 150 mM sodium chloride, 5 mM EDTA and 0.02% sodium azide as a preservative) for 5 min at room temperature (RT) followed by overnight incubation with anti-E-cadherin or mouse polyclonal sera against ZIKV at 4 °C. Following which the cells were incubated with Alexa flour-conjugated secondary antibodies for 1 h at RT. The cells were then incubated with Hoechst 33258 in PBS at 10 μg/ml for 1 h at RT to stain the nuclei. Membranes were then cut out using a scalpel blade, mounted on glass slides with Prolong anti-fade reagent (Invitrogen) and covered with cover-slips and left to dry overnight in dark at 4 °C. The cells were visualized using an Eclipse Ti confocal microscope (Nikon) and NIS Elements Imaging Software.

### RNA extraction and RT PCR

Trizol was added to cell monolayer (1 ml/ well) samples in the appropriate amount and allowed to homogenize from 10 min at room temperature (RT). RNA was extracted as per the manufacturer’s protocol and quantified using Verso-1 step (Verso SYBR Green one-step qRT-PCR kit) using specific primers (Additional file 1: Table S1).

### SDS-PAGE and western blotting

Infected cells were harvested in RIPA lysis buffer supplemented with LDS buffer (Invitrogen) and boiled in reducing sample buffer for 10 min at 80 °C. The samples were then subjected to reducing Novex 4–12% Bis-Tris gel electrophoresis Separated proteins were electroblotted to PVDF membranes by using the NOVEX Xcell Blot II module and probed with Zika virus M protein antibody (GeneTex) or GAPDH antibody (ThermoFisher) or Axl (Cell Signaling Technology).

### Entry assay

4 × 10^4^ Caco-2 cells were seeded onto 6.5 mm diameter, 1 mm pore size polycarbonate membrane trans-wells (Costar) and fresh medium was added at 2 d intervals. At Day 6 post-seeding, the cells were verified to have around 100 Ω resistance before being used for infection. 50 μl of ZIKV suspension at a concentration of 3 pfu/ cell was added either apically or basolaterally, and incubated for 1 h at 37 °C, and washed three times with PBS, followed by addition of 2% fetal bovine serum medium and incubation at 37 °C. Cells were harvested in Trizol reagent and RIPA buffer for RNA and protein analysis respectively at appropriate lysis buffer at indicated time points, and ZIKV M was detected by sodium dodecyl sulfate-polyacrylamide gel electrophoresis (SDS-PAGE) and qRT-PCR.

### Egress assay

Polarized Caco-2 cells (Day 6 post seeding) were infected with ZIKV-PR (3 pfu/ cell) either apically or basolaterally, and incubated for 1 h at 37 °C, and washed three times with PBS, followed by addition of 2% fetal bovine serum medium and incubation at 37 °C. Following 48 h incubation, the supernatants from the top and bottom were harvested and the volume was equalized by adding appropriate amount of MEM supplemented with 2% FBS. The supernatants were then used as inoculum to infect Vero cells. For this purpose, 6 well plates were seeded at a density of 0.3 × 10^6^ per well. The ZIKV supernatant was added onto the cells and incubated for 1 h at 37 °C, followed by removal of inoculum and addition of 2% fetal bovine serum medium and incubated at 37 °C. At 48 h post infection, the cells were harvested in RIPA buffer for protein analysis. Alternatively, the supernatants were spiked with equal concentration of MS-2 phage for normalization and harvested in Trizol LS for qPCR analysis as previously described [29].

### Fluorescein leakage test

Permeability of the monolayer was assessed by measuring leakage of fluorescein dye across the monolayer as previously described [30, 31]. The cells were infected with ZIKV as described above. At 48 hpi cells were washed twice with HBSS. FITC-dextran was added to the upper chamber of ZIKV-infected polarized Caco-2 cells at 48 hpi and incubated for 2 h at 37 °C. Non-polarized cells were used as a control. Levels in the lower chamber were detected by measuring the absorbance at 530 nm.

## Results

### Polarized Caco-2 cells support ZIKV replication

**Fig. 1:**
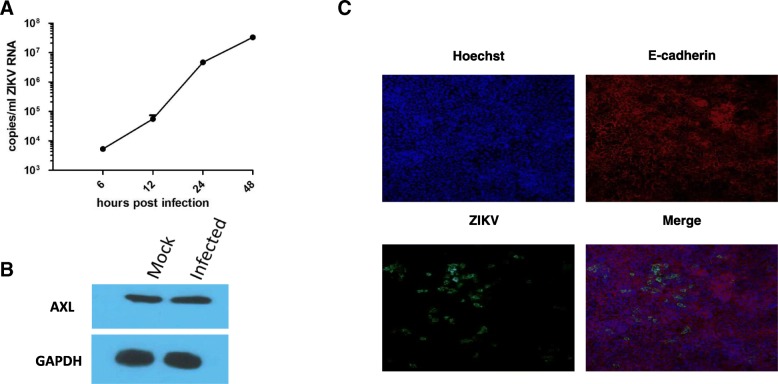
Polarized Caco-2 cells are susceptible to ZIKV infection**. a** Polarized Caco-2 cells were infected with 3 pfu/cell ZIKV-PR, and analyzed for cellular ZIKV RNA levels by qRT-PCR at indicated times (**b**) Polarized Caco-2 cells express Axl as shown by western blot analysis. GAPDH was used as a loading control. **c** Caco-2 monolayers were infected apically with ZIKV and fixed in 10% neutral buffered formalin solution and stained for E-cadherin (red) and ZIKV (green). Nuclei were detected by Hoechst stain (blue)

Caco-2 cells are a well-established model systems for the study of polarized epithelial cells. We have previously characterized a polarized Caco-2 cell model that can be used to elucidate viral infection kinetics in a polarized system [32]. Reports suggest that Caco-2 cells support ZIKV replication, but specific data in polarized cells are lacking [33]. Since polarity is known to affect virus infection efficiency, we first tested the ability of ZIKV-PR to infect polarized Caco-2 cells [34]. Caco-2 cells seeded for 6 days showing around 100 Ω resistance were infected with ZIKV-PR at a concentration of 3pfu/ cell. qRT-PCR analysis showed that ZIKV RNA increase from 6 to 48 hpi (Fig. 1a) indicating a productive infection.

We also confirmed that AXL, the putative receptor for ZIKV, is expressed in the Caco-2 monolayer using western blot analysis. (Fig. 1b). Further, polarized monolayers were infected with ZIKV-PR from the apical surface. The infected monolayers were fixed 24 h post-infection (hpi) and stained for E-cadherin and ZIKV. (Fig. 1c). E-cadherin expression on the cell periphery showed the cell monolayer was intact, and ZIKV positive cells could be seen in at 24hpi. Taken together, this data show that polarized Caco-2 cells are susceptible to ZIKV infection and can be used as a model to study ZIKV pathogenesis in polarized cells.

### ZIKV preferentially enters cells through the apical route

**Fig. 2:**
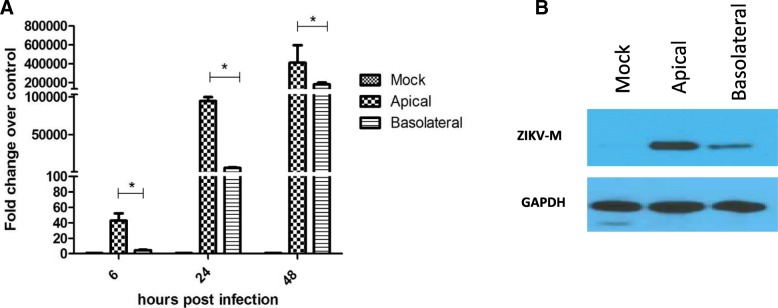
Apical infection of ZIKV is more efficient in Caco-2 cells**. a** Caco-2 cells infected at 3 pfu/ cell were assessed for ZIKV RNA expression at 6, 24, and 48 hpi, using SYBR-green qPCR assay and normalized to GAPDH expression. Results are expressed in mean ± SD calculated from three independent experiments. Data was analyzed using one way ANOVA *p < 0.05. **b** Caco-2 cells infected at 3 pfu/ cell were assessed for ZIKV-M protein expression at 48 hpi by Western Blot analysis. GAPDH was used as a loading reference

Epithelial polarization causes a cell asymmetry, with cells preferentially expressing proteins on one membrane surface or the other. We hypothesized that this unequal protein distribution in polarized cells would affect virus entry. To determine if ZIKV infection occurs via a preferred route into polarized cells, Caco-2 cells were grown on semipermeable Transwell filter inserts until polarity and infected either apically or basolaterally with ZIKV-PR at concentration of 3 pfu/ cell. To measure infection, cell monolayers were lysed at 6, 24, and 48 hpi to harvest viral RNA, while protein was harvested 48 hpi. ZIKV RNA was measured by one step qRT-PCR, and the samples were normalized to the housekeeping gene glyceraldehyde 3-phosphate dehydrogenase (GAPDH). Analysis of viral RNA (Fig. 2a) demonstrated that cells infected from the apical surface showed significantly higher expression of viral RNA at all time-points, suggesting that more susceptible to ZIKV at the apical surface. Additionally, greater ZIKV-M protein could be detected at 48 hpi (Fig. 2b). These results suggest that ZIKV infection occurs preferentially through the apical membrane.

### ZIKV egress occurs through the basolateral route

Viruses which bud apically tend to cause localized infections while basolaterally budding viruses are more likely to cause systemic infections [35]. Though apically budding viruses (like influenza) might still cause systemic infections, viruses that bud basolaterally may more easily reach the underlying tissues and establish faster systemic infections [36]. Viral budding at specific membrane locations requires the transport of all structural viral components to these specific membrane domains. Accordingly, viruses have evolved mechanisms for the polarized transport of their proteins to the apical or basolateral surfaces of epithelial cells.

**Fig. 3:**
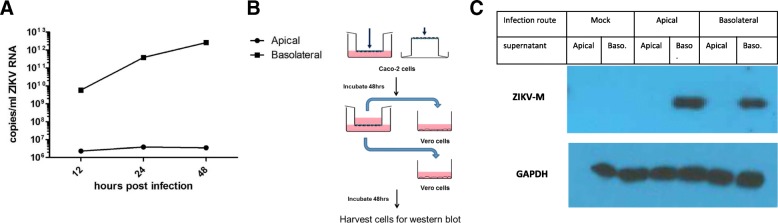
ZIKV release occurs through the basolateral side in Caco-2 cells**. a** Apical and basal media was harvested at different time points SYBR-green qPCR assay and normalized to MS-2 expression. Results are expressed in mean ± SD calculated from three independent experiments. **b** Caco-2 cells were grown on semipermeable Transwell supports and infected either apically or basolaterally at 3 pfu/ cell. After 1 h of infection at 37 °C, the cells were washed with 5X volume of sterile PBS. At 48 hpi, supernatants from the top and bottom chambers were used to infect Vero cells seeded in 6-well plates. Extra media was added to the apical samples to normalize volumes of the two samples. **c** Cells were lysed in RIPA buffer, and used for Western Blot analysis after 48 h incubation

To determine whether ZIKV shows a preference for the site of release in polarized epithelial cells, Caco-2 cells grown on Transwell filter inserts were infected either apically or basolaterally with ZIKV-PR at a concentration of 3 pfu/ cell. Supernatants were collected at 48 hpi from the apical and basolateral compartments. Since the virus in supernatant was below the limit of detection by western blot analysis, it used as inoculum to infect Vero-E6 cells, incubated for a further 48 h (Fig. 3a). The cells were then harvested in RIPA buffer and tested for the presence of ZIKV-M. Western blot analysis showed expression of ZIKV-M exclusively in cells infected with the basolateral supernatants, from cells infected from either apical or basolateral routes (Fig. 3b). Further, viral release into apical and basal media was determined by measuring ZIKV RNA in the basal supernatant (Fig. 3c). Viral RNA is detected in the basal supernatant as early as 12 hpi, and increases over time. Apical supernatant also tested positive for ZIKV RNA, however, it remained steady over time, indicating its likely leftover inoculum from the cells, and virus release seems to occur basolaterally in Caco-2 cells. Taken together, these results indicate that release of ZIKV occurs primarily at the basolateral surface.

### ZIKV infection does not affect epithelial barrier function

Tight junctions play a major role in maintaining the barrier function in epithelial cells [15]. They form the border between the apical and basolateral plasma-membrane domains and are linked to the machinery that controls apico-basal polarization [37]. A number of flaviviruses, including Dengue virus (DENV) West Nile virus (WNV) and Japanese Encephalitis (JEV) have been shown to induce degradation of tight junction proteins to disrupt the tight junctional barrier [27, 38, 39]. Additionally, ZIKV has been shown to affect barrier permeability in retinal pigment epithelial cells day 7 post infection [10]. Since our results showed the virus buds from the basal route, we wanted to investigate the possibility that ZIKV causes disruption of the epithelial barrier and the virus passively translocates to the bottom chamber rather than actively being directed at the basal membrane.

**Fig. 4:**
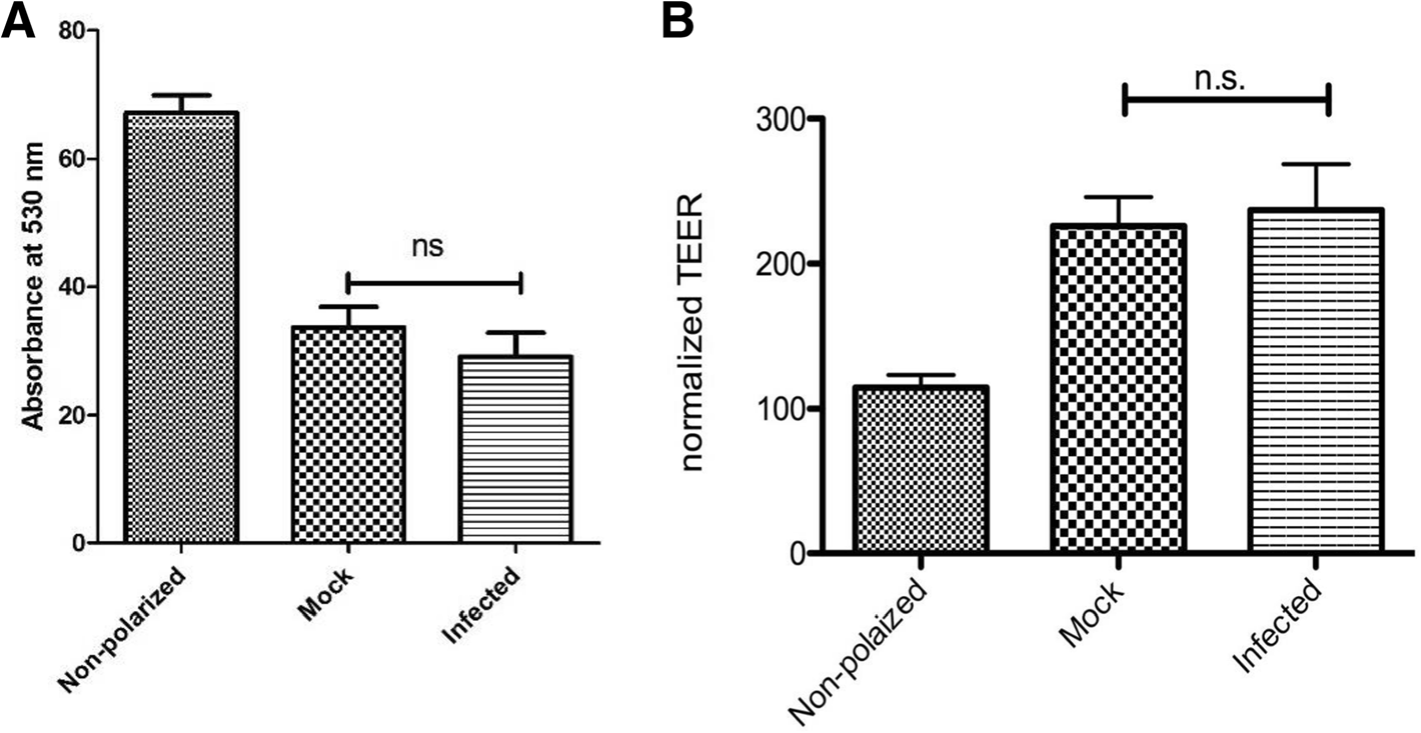
ZIKV translocation occurs without disruption of cell permeability**. a** Cellular permeability was assayed by using FITC-dextran (molecular weight 70,000) dissolved in Hanks Balanced Salt Solution (HBSS). FITC-dextran was added to the upper chamber of ZIKV-infected polarized Caco-2 cells at 48 hpi and incubated for 2 h at 37 °C. Non-polarized cells were used as a control. Levels in the lower chamber were detected by measuring the absorbance at 530 nm. All results were normalized to an HBSS blank. Results are expressed as mean ± SD calculated over three independent experiments. Data was analyzed using the Mann-Whitney test *p < 0.05. **b** Caco-2 cells were grown on semipermeable Transwell supports and TEER of ZIKV- or mock-infected monolayer was measured 48hpi. Non-polarized cells were used as a control. Results in Ω are mean values of triplicates. Data was analyzed using one-way ANOVA n.s. > 0.05

To investigate the general impact of ZIKV on epithelial barrier function, paracellular permeability of Caco-2 cells was measured. The cells grown on Transwell inserts were infected apically with ZIKV-PR at A concentration of 3 pfu/ cell (Fig. 4a). At 48 hpi, the impact of infection on cellular permeability was assessed by the rate at which water soluble fluorescein isothiocyanate (FITC)-dextran was transported across the epithelium to the bottom chamber upon addition at the top chamber. Calorimetric measurement of FITC-dextran after an incubation of 2 h showed that until 48 hpi, the virus does not impact the cellular barrier. To confirm this finding, we also examined the impact of infection on TEER. Measurement of TEER 48hpi showed no statistical difference between the virus or mock infected monolayer. Together, these data indicate that basolateral release of the virus is not because of barrier disruption but a selective process. Interestingly, there are reports observing a reduction in tight junction protein ZO-1, 11 days post-infection, indicating that like other flaviviruses, ZIKV may cause degradation of tight junction proteins, but it may occur at later stages of infection [10].

## Discussion

Though there have been reports in literature about polarized release of ZIKV, to our knowledge, this is the first study that evaluates the ability of ZIKV to infect polarized epithelial cells through different membranes. Our data show that polarized Caco-2 cells are susceptible to ZIKV infection. Caco-2 are preferentially infected from the apical surface and vectorially released from the basolateral route without a change in cellular permeability.

ZIKV is known to use Axl as an entry receptor, in microglia, astrocytes and endothelial cells [7, 40]. However, there are no reports in literature examining polarized Caco-2 cells. Our efforts to establish polarity were also limited my technical difficulties, as we were unable to find an antibody that was suitable for confocal microscopy. Further studies are thus needed to examine the occurrence of surface selective expression of the protein in polarized cells. However, literature shows that this receptors alone may not be the limiting factor for entry. In the case of adeno-associated virus, endosomal processing, rather than receptor availability can be important [41]. Another possibility is that factors which stabilize virus adsorption may be asymmetrically present on the two surfaces. For instance, several studies have indicated that flaviviruses make initial contact with the host cell by binding to glycosaminoglycans (GAGs) [42–44]. Interestingly, certain GAGs are known to be differentially distributed in polarized cells [45].

Our data show that ZIKV buds preferentially through the basolateral surface. This is in agreement with recent studies in both epithelial and endothelial cells, which show basolateral budding [10, 46]. Though the exact viral factors involved in polarized sorting are unclear, it is likely that the signal comprises of tyrosin-based or dileucine based motif occurring on prM and E, since both are known to play a critical role in virus budding [13, 47, 48]. The microtubule network may also be involved as in the case of WNV polarized budding [19]. In contrast to other reports however, our reports show that ZIKV does not cause a disruption in cell permeability. Both cell permeability and cell junction architecture remained unaffected during ZIKV infection. This may be because of the cell system used, as other studies used retinal pigment epithelial (RPE), or endothelial cells while our study uses Caco-2 cells. Further, our time window of measurement was upto 48hpi, which is shorter than other reports which go upto 7 days. Therefore, ZIKV may indeed cause cell barrier disruption in Caco-2 cells, but may need a longer period for infection to induce these changes.

Taken together, we show that ZIKV egress occurs in a directed manner, and is not passive translocation due to tight junction disruption as seen in the case of tick-borne encephalitis virus (TBEV) [38].

It is thought that the ability of a virus to bud apically or basolaterally from epithelial cells plays an important role in the pathogenicity and invasiveness of the virus [49]. Therefore, the ability to ZIKV to cross the epithelial barrier without being reliant on cell barrier disruption may provide an advantage during viral dissemination in the host.

## Conclusions

Our data show that polarized epithelial cells are susceptible to ZIKV infection. The virus enters preferentially through the apical side and buds selectively through the basolateral membrane. Data from permeability assays and electron microscopy indicate that the virus is actually translocating transcellularly rather than paracellular manner, and ZIKV does not need disruption of TJ proteins to cross the epithelial barrier. This translocation across the epithelial membrane may facilitate delivery of virions to sub-epithelial layer and aid in ZIKV dissemination through the host.

## Additional file

Additional file 1: **Table S1** List of primer sequences. (DOCX 12 kb)

## Data Availability

All data generated or analyzed during this study are included in this published article.

## Abbreviations

DENV: Dengue virus
FBS: Fetal bovine serum
GAPDH: Glyceraldehyde 3-phosphate dehydrogenase
hpi: Hours post infection
M: Matrix protein
MEM: Minimum Essential Medium
qPCR: Quantitative polymerase chain reaction
RT: Room temperature
TBEV: Tick-borne Encephalitis virus
TEER: Transepithelial electrical resistance
TJ: Tight junctions
WNV: West Nile virus
ZIKV: Zika virus
