# Supplementary material for: Directional entry and release of Zika virus from polarized epithelial cells
Source: Virol J. 2019 Aug 8;16:99. doi: 10.1186/s12985-019-1200-2 (PMC6688342; doi:10.1186/s12985-019-1200-2)
Supplement: Supplementary file 1 — Table S1 List of primer sequences. (DOCX 12 kb) [file 12985_2019_1200_MOESM1_ESM.docx]

**Supporting information**

**List of primer sequences.**

| Primer | Sequence (5’-3’) |
| --- | --- |
| ZIKV FWD | GAGTGTGATCCAGCCGTTATT |
| ZIKV REV | CAGCCTCCATGTGTCATTCT |
| GAPDH FWD | CAACTCACCTCTTGGGATGAAG |
| GAPDH REV | CCTGGTTCAGTTTGGAGTCTATG |
| MS2 FWD | TGCTCGCGGATACCCG |
| MS2 REV | AACTTGCGTTCTCGAGCGAT |
